# Targeting the IKZF1/BCL-2 axis as a novel therapeutic strategy for treating acute T-cell lymphoblastic leukemia

**DOI:** 10.1080/15384047.2025.2457777

**Published:** 2025-01-25

**Authors:** Juan Li, Chunmei Ye, Hui Li, Jun Li

**Affiliations:** a Department of Hematology, Taixing People’s Hospital Affiliated to Yangzhou University, Taixing, China; b Institute of Hematology, Affiliated hospital of Yangzhou University, Taixing, China

**Keywords:** IKZF1, BCL-2, venetoclax, T-cell acute lymphoblastic leukemia

## Abstract

**Objectives:**

Acute T-cell lymphoblastic leukemia (T-ALL) is a severe hematologic malignancy with limited treatment options and poor long-term survival. This study explores the role of IKZF1 in regulating BCL-2 expression in T-ALL.

**Methods:**

CUT&Tag and CUT&Run assays were employed to assess IKZF1 binding to the BCL-2 promoter. IKZF1 overexpression and knockdown experiments were performed in T-ALL cell lines. The effects of CX-4945 and venetoclax, alone and in combination, were evaluated in vitro and in vivo T-ALL models.

**Results:**

CUT&Tag sequencing identified IKZF1 binding to the BCL-2 promoter, establishing it as a transcriptional repressor. Functional assays demonstrated that IKZF1 overexpression reduced BCL-2 mRNA levels and increased repressive histone marks at the BCL-2 promoter, while IKZF1 knockdown led to elevated BCL-2 expression. CX-4945, a CK2 inhibitor, could reduced BCL-2 levels in T-ALL cells. Notably, knockdown of IKZF1 partially rescued the CX-4945-induced repression of BCL-2. These results underscore the CK2-IKZF1 signaling axis as a key regulator of BCL-2 expression. In vitro, CX-4945 enhanced the cytotoxicity of venetoclax, with the combination showing significant synergistic effects and increased apoptosis in T-ALL cell lines. In vivo studies with cell line-derived xenograft (CDX) and patient-derived xenograft (PDX) models demonstrated that CX-4945 and venetoclax combined therapy provided superior therapeutic efficacy, reducing tumor burden and prolonging survival compared to single-agent treatments.

**Conclusions:**

IKZF1 represses BCL-2 in T-ALL, and targeting the CK2-IKZF1 axis with CX-4945 and venetoclax offers a promising therapeutic strategy, showing enhanced efficacy and potential as a novel treatment approach for T-ALL.

## 1. Introduction

Acute T-cell lymphoblastic leukemia (T-ALL) is a highly aggressive hematologic malignancy that poses a substantial threat to human health.^1,2^ It accounts for approximately 15% of childhood acute lymphoblastic leukemia (ALL) cases and 25% of adult ALL cases,^1,3^ characterized by a high relapse rate and poor prognosis. For adult T-ALL patients, the 5-year survival rate is around 50%, with long-term survival after relapse dropping to less than 10%.^4,5^ Current treatment strategies primarily rely on conventional chemotherapy and hematopoietic stem cell transplantation, but progress in developing targeted therapies has been slow due to a lack of new therapeutic targets.

Casein kinase II (CK2) is a highly conserved serine/threonine protein kinase that is ubiquitously expressed in eukaryotic cells.^6–8^ It is composed of two catalytic subunits (CK2α and CK2α’) and two regulatory subunits (CK2β).^9,10^ CK2 plays a pivotal role in a myriad of biological processes, including cell proliferation, apoptosis, DNA damage repair, and cell cycle regulation, by phosphorylating its substrates and modulating the activity of the signaling pathways they participate in.^10,11^ Elevated CK2 activity is notably associated with increased tumor proliferation, invasiveness, and drug resistance, suggesting that CK2, as a significant oncogene, contributes to the initiation and progression of various human cancers.^9,10,12^ Recent research has indicated that targeting CK2 could represent a novel therapeutic strategy in human cancers, including T-ALL.^8,11,13,14^

CX-4945 (molecular weight: 349.77) is a small molecule inhibitor of CK2,^15^ capable of diminishing the catalytic activity of CK2 by competitively binding to the catalytic subunits CK2α and CK2α’, thereby inhibiting its function.^11,15,16^ CX-4945 exhibits high selectivity for CK2 and exerts minimal inhibitory effects on other kinases.^9^ Prior studies have revealed that aberrantly elevated CK2 activity can diminish the function of the crucial tumor suppressor protein IKAROS (IKZF1) through phosphorylation and inactivation in ALL.^9,17,18^ CX-4945 has been shown to effectively suppress CK2 activity, restore the tumor suppressor capabilities of IKAROS, induce cell cycle arrest, and curb the proliferation of leukemia cells.^9,10,17,18^

In this study, we show that IKZF1 represses the transcription of *BCL-2* in T-ALL. We also explore the effects of combining the BCL-2 inhibitor venetoclax with CX-4945 in T-ALL. Through *in vitro* and *in vivo* experiments, we assess the anti-leukemic efficacy of the two drugs, investigating potential synergistic effects. Our results aim to support future clinical applications of this combination therapy in T-ALL.

## 2. Materials and methods

### 2.1. Cell lines and agents

The T-ALL cell lines CEM, MOLT-4 and Jurkat cells were purchased from American Type Culture Collection (ATCC, USA) and were grown in RPMI-1640 (Hyclone, Shanghai, China) supplemented with 10% heat-inactivated Fetal bovine serum (FBS) (Gibco, Beijing, China), 1% penicillin-streptomycin and 1% L-glutamine, and in a 5% CO2 atmosphere at 37°C.

CK2 inhibitor CX-4945 was purchased from MedChemExpress (HY-50855, New Jersey, USA), dissolved in a 100 μM RPMI 1640 solution, and stored at −20°C. Venetoclax was purchased from MedChemExpress (HY-15531, New Jersey, USA), and the 10 mm stock solution is dissolved in DMSO and stored at −20°C.

### 2.2. Cell counting kit-8 (CCK-8) cell proliferation assay

Cells were seeded with 96-well plates at a density of 1 × 10^4^ cells per well, as previously described. Various dosages of CX-4945 (0–20 μM) or venetoclax (0–16 μM) and DMSO vehicle control were added to the 96-well plate and then incubated for 48 hours. For the CCK-8 assay, cells were added with the 10 μL solution of CCK-8 (Dojindo, Kumamoto, Japan) and incubated at 37°C for 4 hours. The absorbance of each well was measured at 450 nm by using a Multiskan Go spectrophotometer (Thermo Fisher Scientific, Inc.). The drug-mediated cell proliferation arrest with the indicated doses was calculated by comparing the absorbance versus that of no treatment control. Each experiment was carried out in triplicate, and each sample was triplicated and assayed per assay.

### 2.3. Colony-forming assay

T-ALL cells were collected and washed after 12 hours of pre-incubation with CX-4945 treatment. Cells were then cultured in methylcellulose medium (Stem Cell Technologies, Canada) at a density of 500 viable cells per well. After 10–14 days, colonies were stained with MTT solution (5 mg/mL) (Solarbio, China). %CFU (colony forming unit) = CFU (experimental group)/CFU (control group) × 100%.

### 2.4. RNA extraction, and quantitative RT-PCR

Total RNA was isolated from cells using the TRIzol reagent (TAKARA, Japan), followed by the indicated treatments. cDNA was generated from 1 μg total RNA using Superscript First-Strand Synthesis System (Invitrogen). RT-PCR was performed using a StepOne Plus real-time PCR machine (Applied Biosystems) with comparative qPCR with SYBR Premix EX TaqII (TAKARA, Japan). Values were normalized to GAPDH, and the relative expression values were determined by the 2^−ΔΔCt^ method. The primers for RT-PCR are listed in **Table S1**.

### 2.5. Cell apoptosis analysis

Cells were collected after drug treatment for 48 hours. Apoptosis assays were performed following the manufacturer’s instructions using the Annexin V- Propidium Iodide (PI) apoptosis detection kit (BD, NO.550474) and analyzed on flow cytometry (Thermo, Attune NxT, USA). Cells were stained with annexin V and PI for 20 minutes at room temperature and analyzed.

### 2.6. CUT&RUN assay kit for PCR/qPCR

Quantitative ChIP-qPCR (qChIP) assays were conducted using the Hyperactive pG-MNase CUT&RUN Assay Kit for PCR/qPCR, employing target-specific antibodies or normal rabbit IgG (Abcam, ab46540) as a control. The antibodies used for qChIP included anti-IKZF1 (A303-516A-T, Bethyl, Montgomery, USA) and anti-H3K4me3 (ab580, Abcam, Waltham, USA). Enrichment of ChIP samples relative to input DNA (fold enrichment) was quantified by qPCR in triplicate to ensure data reliability.

### 2.7. Western blotting

Cell lysates from cells treated with C×4945for 48 hours were prepared on ice in lysis buffer [50 nM Tris-HCL (pH 7.4), 150 mm NaCl, 1% NP-40, 0.1% SDS], and total cell lysate was extracted using column cell protein extraction kit (EpiZyme, China) following the manufacturer’s protocol. SDS-PAGE gel (Beyotime Biotechnology, China) was used to isolate proteins, the following transfer to PVDF membranes (Millipore, USA). The blots were then incubated with the following primary antibodies: anti-BCL-2 (Abcam, No. ab32124), anti-GAPDH (Proteintech, NO.1E6D9).

### 2.8. Luciferase assays of promoter activity

The promoter regions (−1 to −500bp) of *BCL2* was cloned into the pGL2 vector (Promega, Madison, USA) respectively. The transient luciferase assay was performed in 293T cells as previously described. Luciferase activities were calculated as fold change relative to values obtained from pGL2 vector-only control cells and expressed as a percentage of pcDNA3.1-*IKZF1* or pcDNA3.1-*BCL2* transfection-induced luciferase activity versus that of pcDNA3.1 vector. All transfection and reporter assays were performed independently with three replicates.

### 2.9. Statistical analysis

Data were presented as the mean ± SD. A t-test was used to identify differences between groups (paired t-test was used for paired groups), and an A-NOVA test was used to compare several groups (>2 groups). The half-maximal inhibitory concentration (IC50) was calculated by GraphPad Prism 8.0. The combination index was calculated by Calcusyn software.

Additional details regarding CUT&Tag assay for sequencing, gene knockdown or overexpression, and construction of xenograft mouse models were found on supplementary materials.

## 3. Results

### 3.1. IKZF1 modulates the BCL-2 expression in transcriptional level

**Figure 1.**
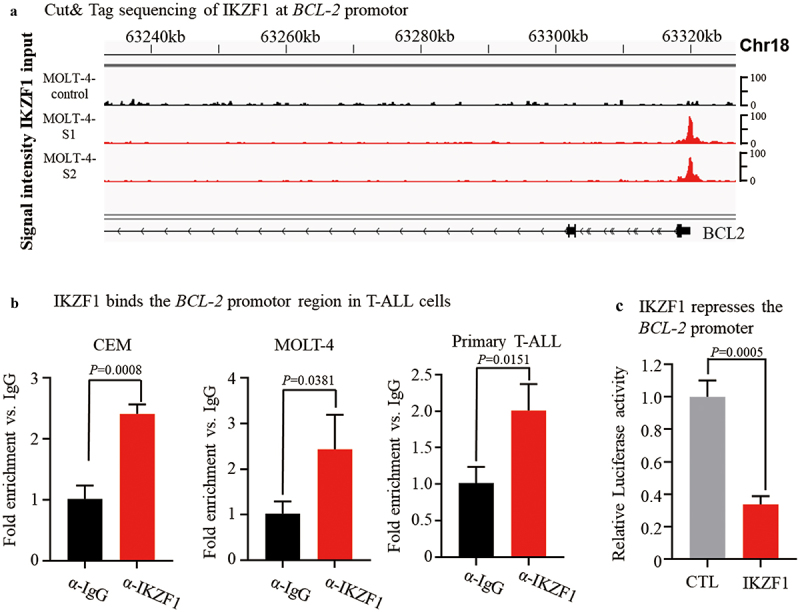
IKZF1 binds the promoter of *BCL-2* gene and suppress *BCL-2* expression. (a) Cut&Tag sequencing identified that IKZF1binds at *BCL-2* promoter region in MOLT-4 cells. (b) Cut&Run for qPCR analysis confirming IKZF1 binding at the promoter in T-ALL cell lines. (c) Luciferase reporter assay following transfection with *IKZF1* plasmids or control vector in 293T cells assessed the activity of the *BCL2* promoter region (−1kb to + 500kb).

We first employed CUT&Tag sequencing to identify the genomic targets of the transcription factor IKZF1 in T-ALL. Our analysis revealed that IKZF1 specifically binds to the promoter region of the *BCL-2* gene in the MOLT-4 cell line (Figure 1a). This finding was further confirmed using CUT&Run followed by qPCR analysis, which demonstrated IKZF1 binding at the *BCL-2* promoter in both CEM (*p* = .0008) and MOLT-4 (*p* = .0381) cell lines, as well as in primary T-ALL cells (*p* = .0151) (Figure 1b).

To assess the functional impact of IKZF1 binding at the *BCL-2* promoter, we performed transient co-transfection assays using a *BCL-2* promoter construct (−1 kb upstream to + 500 bp downstream of the transcription start site). Co-transfection of *IKZF1* with this promoter construct significantly reduced luciferase activity (*p* = .0005), as shown in Figure 1c.

These findings indicate that IKZF1 suppresses *BCL-2* transcription by binding directly to its promoter.

### 3.2. IKZF1 repress the transcription of BCL-2

**Figure 2.**
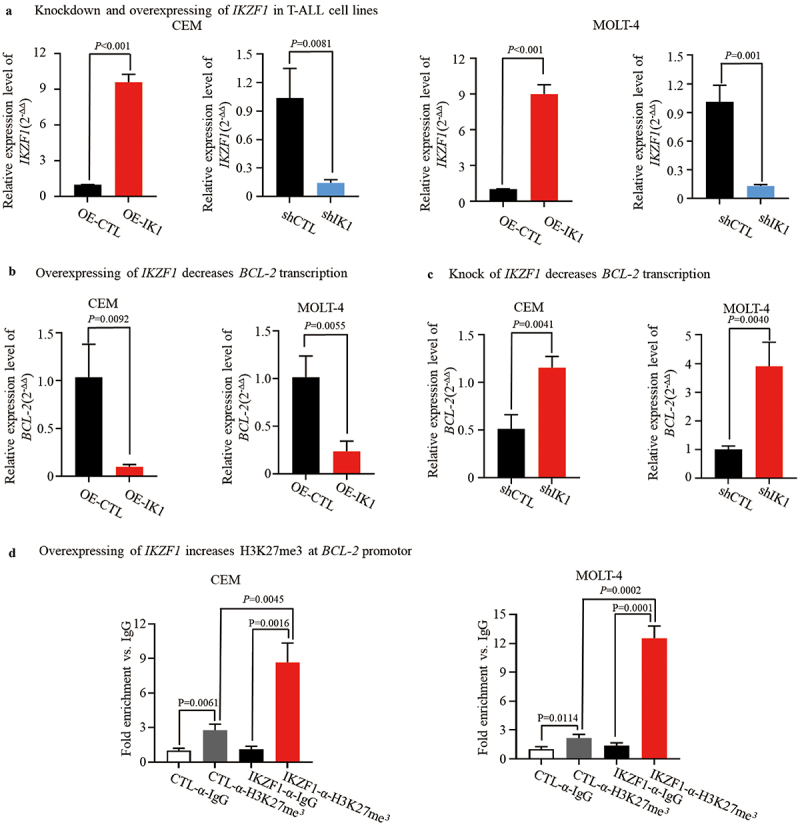
IKZF1 modulates the transcription of *BCL-2* gene via chromatin remodeling. (a) *IKZF1* was knockdown and overexpressed in T-ALL cells. (b) CEM and MOLT-4 T-ALL cell lines were transduced to express *IKZF1* (OE-IK1) or with empty vector (OE-CTL). Relative expression of *BCL-2* was assessed by qRT-pcr. (c) CEM and MOLT-4 T-ALL cell lines were treated with *IKZF1* shRNA (shIK1) or control shRNA (shCTL). The relative expression of *BCL-2* was assessed by qRT-pcr. (d) Cut&Run for qPCR analysis showed the presence of H3K27me^3^ marks at the *BCL-2* promoter region in CEM and MOLT-4 T-ALL cells with *IKZF1* overexpressing. Data were presented as mean ± SD of combined values from three independent experiments.

We investigated the role of IKZF1 in the transcriptional regulation of *BCL-2*. *IKZF1* was effectively knocked down and overexpressed, respectively in CEM (Figure 2a) and MOLT-4 (Figure 2a) cells. Overexpression of *IKZF1* through retroviral transduction significantly decreased *BCL-2* mRNA levels in CEM (*p* = .0092) and MOLT-4 (*p* = .0055) cell lines, as shown in Figure 2b. Conversely, knockdown of *IKZF1* using shRNA resulted in a significant upregulation of *BCL-2* expression in both CEM (*p* = .0041) and MOLT-4 (*p* = .0040) cells (Figure 2c). Notably, *IKZF1* overexpression was associated with increased deposition of the repressive histone mark H3K27me3 at the *BCL-2* promoter in CEM (IKZF1-α-H3K27me3 vs. CTL-α-H3K27me3, *p* = .0045) and MOLT-4 (IKZF1-α-H3K27me3 vs. CTL-α-H3K27me3, *p* = .0002) cell lines (Figure 2d).

These findings demonstrate that IKZF1 acts as a transcriptional repressor of *BCL-2* in T-ALL by promoting repressive chromatin formation (H3K27me3) at the *BCL-2* promoter.

### 3.3. CX-4945 restored IKZF1-mediated repression of BCL-2

CK2 reduces the DNA-binding affinity of IKZF1 via phosphorylation, and pharmacological inhibition of CK2 can restore IKZF1 function. In this study, we investigated the effect of CK2 inhibition by CX-4945 on *BCL-2* expression in T-ALL cell lines.

**Figure 3.**
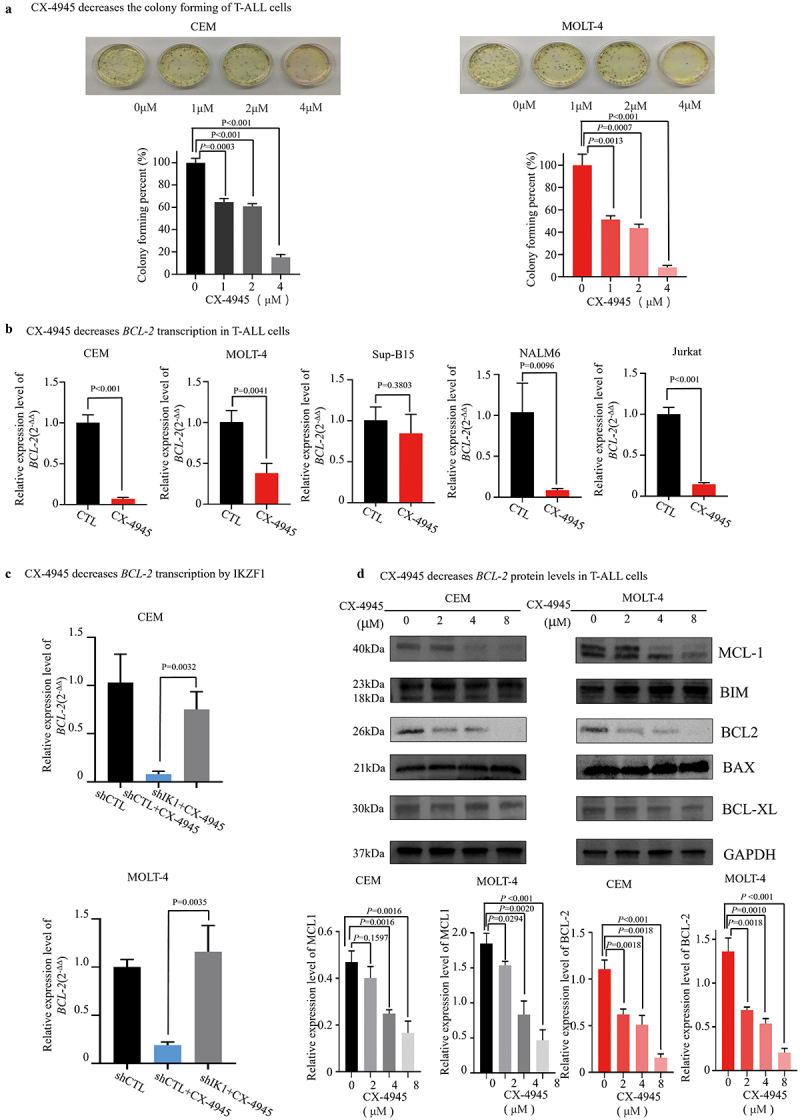
CX-4945 represses the transcription of *BCL-2*. (a) 0, 2, 4, 8 μM CX-4945 treatment in CEM and MOLT-4 T-ALL cell lines reduced the colony forming. (b) Function restoration of IKZF1 by 5 μM CX-4945 for 2 days in CEM, MOLT-4, sup-B15, NALM6 and Jurkat cell lines reduced the transcription of *BCL-2*. Relative expression of BCL-2 was assessed by qRT-pcr. (c) Effect of *IKZF1* knockdown (sh*IKZF1*) or scramble shRNA control (shCTL) on changes in BCL-2 gene expression induced by 5 μM CX-4945 treatment for 2 days. (d) 0, 2, 4, 8 μM CX-4945 treatment in CEM and MOLT-4 T-ALL cell lines for 2 days reduced the protein levels of BCL-2 and MCL-1. Data were presented as mean ± SD of combined values from three independent experiments.

Firstly, our findings showed that co-treatment with increasing dosages of CX-4945 in T-ALL cells resulted in significantly reduction of CSF (Figure 3a). To assess whether IKZF1 plays a pivotal role in CK2-mediated regulation of *BCL-2*, we treated CEM and MOLT-4 cells with CX-4945 (4 μM). This treatment resulted in a marked reduction in *BCL-2* expression. Inhibiting CK2 with CX-4945 significantly reduced *BCL-2* transcription in both CEM (*p* < .001) (Figure 3b) and MOLT-4 (*p* = .0041) (Figure 3b) cell lines. Notably, knockdown of *IKZF1* using shRNA partially rescued the CX-4945-induced repression of *BCL-2* in both CEM (shIK1+CX-4945 vs. shCTL+CX-4945, *p* = .0032) and MOLT-4 (shIK1+CX-4945 vs. shCTL+CX-4945, *p* = .0035) cell lines (Figure 3c), indicating that IKZF1 is a key mediator in this pathway.

Western blot analysis confirmed that increasing doses of CX-4945 (0, 2, 4, 8 μM) led to a corresponding decrease in BCL-2 protein levels in T-ALL cells (Figure 3d). Interestingly, our findings also indicated that in T-ALL cells treated with CX-4945, the protein level of MCL-1 decreases with increasing concentrations of CX-4945, while the levels of BCL-XL, BIM, and BAX remain relatively stable. This suggests that the reduction in MCL-1 expression, in addition to the decrease in BCL-2 protein, may contribute to the synergistic anti-T-ALL effects observed with BCL-2 inhibitors.

**Figure 4.**
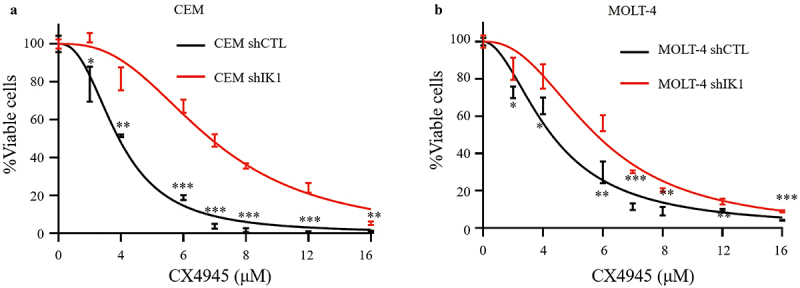
Knockdown of *IKZF1* attenuates the CX-4945 mediated cell proliferation arrest. Knockdown of IKZF1 mitigates the inhibitory effect of CX-4945 on cell proliferation in both CEM (a) and MOLT-4 (b) cell lines.

In addition, knockdown of *IKZF1* attenuated the CX-4945-induced cell proliferation arrest in both CEM (Figure 4a) and MOLT-4 (Figure 4b) cell lines.

These results suggest that *BCL-2* expression is regulated via the CK2-IKZF1 signaling axis, with IKZF1 alterations modulating *BCL-2* levels.

### 3.4. CX-4945 synergize with venetoclax in the treatment of T-ALL cells in vitro

We further explored the effects of CX-4945 and venetoclax on cell proliferation arrest and their potential synergistic cytotoxicity in T-ALL cells. To evaluate this, we compared the cytotoxic impact of the CX-4945 and venetoclax combination therapy against single-agent treatments *in vitro* using two human T-ALL cell lines, CEM and MOLT-4.

**Figure 5.**
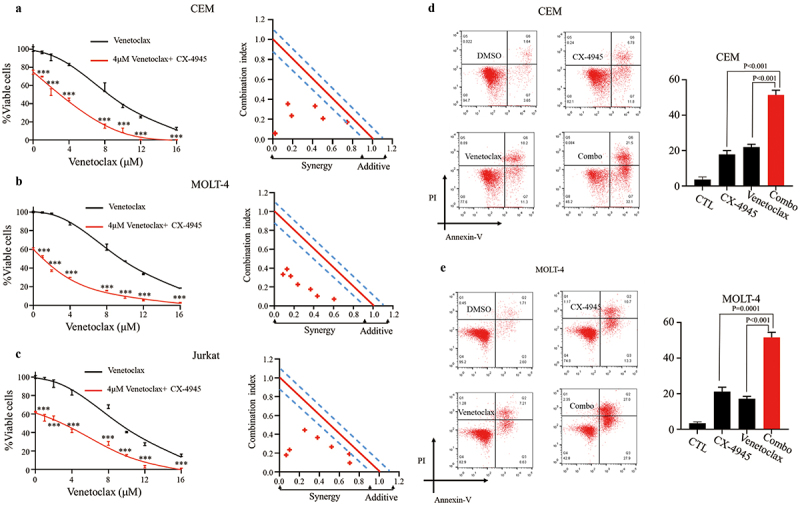
CX-4945 plus venetoclax exerts synergistic effect on cellular proliferation and cellular apoptosis. Cellular proliferation arrest analysis and synergistic effect analysis of 5 μM CX-4945 plus venetoclax for 48 h on CEM (a), MOLT-4 (b) and Jurkat (c) cell lines. Cellular apoptosis effect analysis of 5 μM CX-4945 plus venetoclax for 48 h on CEM (d) and MOLT-4 (e) cell lines. Cellular proliferation was measured by CCK-8 assay. Apoptotic effect was measured by flow cytometry. Synergistic analysis was performed using calcusyn, Y axis is the combination index (CI) value. *p < .05, **p < .01, ***p < .001.

CCK-8 assays revealed that CX-4945 (4 μM) significantly enhanced venetoclax-induced cytotoxicity across a range of concentrations (1, 2, 4, 8, 10, 12, 16 μM) (Figure 5a–c). CalcuSyn analysis further confirmed a notable synergistic effect of the combination therapy in CEM (Figure 5a), MOLT-4 (Figure 5b), and Jurkat (Figure 5c) cells.

To further investigate the synergy between CX-4945 (6 μM) and venetoclax (8 μM) in inducing apoptosis, we analyzed the apoptotic response in T-ALL cells. The combination treatment produced a significant pro-apoptotic effect in both CEM (Combo vs. CX-4945: 51.49 ± 2.63% vs. 17.83 ± 2.19%, *p* < .001; Combo vs. Venetoclax: 51.49 ± 2.63% vs. 21.97 ± 1.60%, *p* < .001) (Figure 5d) and MOLT-4 (Combo vs. CX-4945: 51.68 ± 2.81% vs. 21.20 ± 2.45%, *p* = .0001; Combo vs. Venetoclax: 51.68 ± 2.81% vs. 17.23 ± 1.29%, *p* < .001) (Figure 5e) cells compared to single-drug treatments.

Together, these findings suggest that the combination of CX-4945 and venetoclax exerts a synergistic cytotoxic effect in T-ALL cells.

### 3.5. CX-4945 augments cytotoxicity of venetoclax in cell line-derived xenograft model

To evaluate the potential synergistic effect of CX-4945 and venetoclax *in vivo*, we assessed their therapeutic efficacy in a cell line-derived xenograft (CDX) model. After engraftment of CEM cells, mice were randomly assigned to four groups: vehicle control, CX-4945, venetoclax, and the combination of CX-4945 plus venetoclax. Upon disease onset (on day 22 after tail vein injection of cells), bone marrow and spleen samples were collected to thoroughly assess tumor burden.

**Figure 6.**
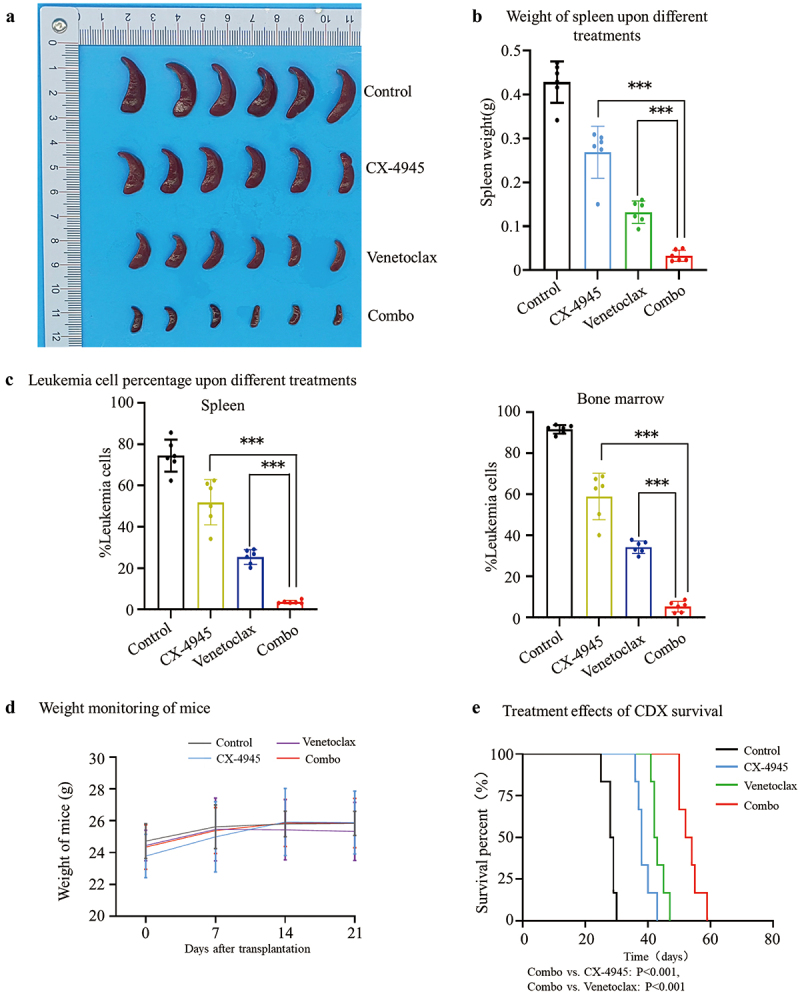
CX-4945 plus venetoclax exerts synergistic effect in cell line derived xenograft mice model. NCG mice were transplanted via tail vein with 2 × 10^5^ cells CEM cells per mouse. After 1 week of, mice were treated with CX-4945 only, venetoclax only, CX-4945 plus venetoclax (Combo) or with vehicle-only (control). Once disease occurred (on day 22 after tail vein injection of cells), the spleens and bone marrow from the mice in each group were harvested for analysis, including the gross appearance of the spleens (a) and the weight of the spleens (b). Spleen (c) and bone marrow cells (d) were stained with human-specific CD45 and CD7 marker antibodies, and the proportion of primitive cells was quantified. The body weight changes of the mice across the groups were monitored throughout the study and statistically analyzed (d). Mice not subjected to tissue collection were observed for survival, and survival curves were generated using the Kaplan-Meier method (e). Differences in survival were analyzed using the chi-square test.

The results demonstrated that the combination of CX-4945 and venetoclax yielded a significantly stronger therapeutic effect in the CDX model compared to either drug alone. Combination treatment notably reduced spleen weight (Combo vs. CX-4945: 0.033 ± 0.013 g vs. 0.27 ± 0.059 g, *p* < .001; Combo vs. Venetoclax: 0.033 ± 0.013 g vs. 0.13 ± 0.025 g, *p* < .001) (Figure 6a). Additionally, the percentage of leukemia cells was significantly decreased in both the spleen (Combo vs. CX-4945: 3.61 ± 0.79% vs. 51.91 ± 10.96%, *p* < .001; Combo vs. Venetoclax: 3.61 ± 0.79% vs. 25.38 ± 3.54%, *p* < .001) (Figure 6b & S1A) and bone marrow (Combo vs. CX-4945: 5.29 ± 2.58% vs. 58.95 ± 11.32%, *p* < .001; Combo vs. Venetoclax: 5.29 ± 2.58% vs. 34.29 ± 3.04%, *p* < .001) (Figure 6c & S1B).

Throughout the study, no significant changes in body weight were observed (*p* > .05) across all treatment groups, indicating minimal toxicity. Kaplan-Meier survival analysis further revealed that the combination of CX-4945 and venetoclax significantly prolonged survival compared to monotherapy (Combo vs. CX-4945: *p* = .0005; Combo vs. Venetoclax: *p* = .0005) (Figure 6d).

Taken together, these findings indicate that the combination of CX-4945 and venetoclax provides a superior therapeutic benefit in the T-ALL CDX model.

### 3.6. CX-4945 augments cytotoxicity of venetoclax in patient-derived xenograft model

We also investigated whether the combination of CX-4945 and venetoclax could produce a strong synergistic effect in a patient-derived xenograft (PDX) model. The PDX model was established using engraftment of primary ETP-ALL (early T-cell precursor acute lymphoblastic leukemia) cells. Following tail vein injection, the mice were divided into four groups: vehicle control, CX-4945, venetoclax, and the combination of CX-4945 plus venetoclax. Upon disease onset (on day 28 after tail vein injection of cells), samples were collected from the bone marrow and spleen to thoroughly assess tumor burden.

**Figure 7.**
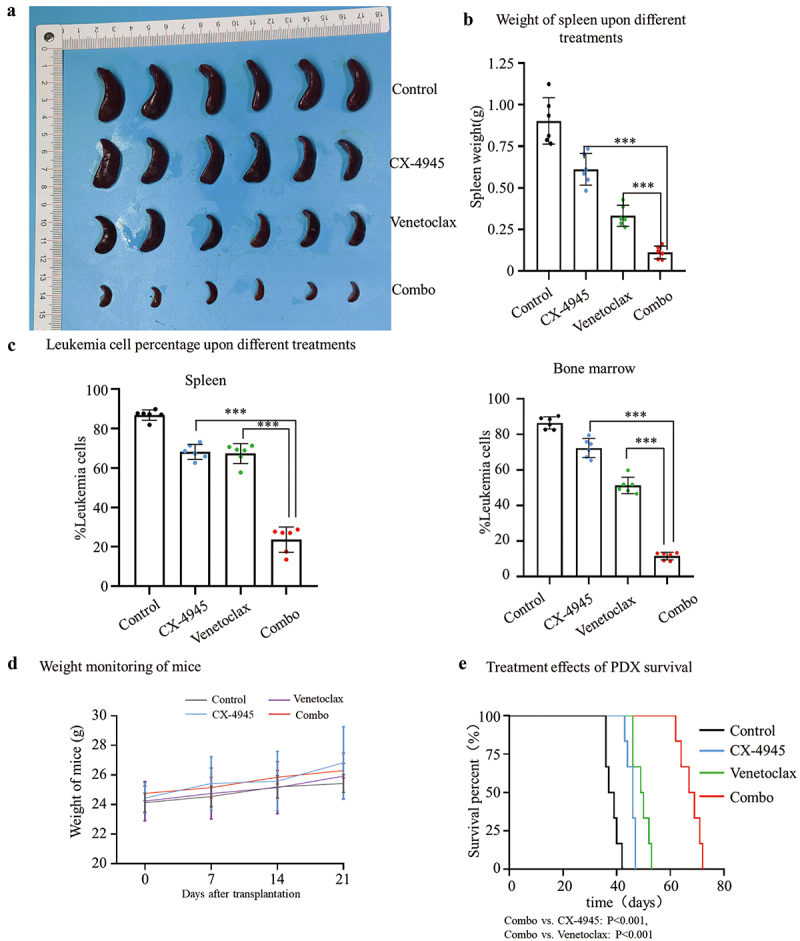
CX-4945 plus venetoclax exerts synergistic effect in patient derived xenograft mice model. NCG mice were injected with 5 × 10^5^ primary ETP T-ALL cells via tail vein injection. A week post-transplantation, treatment commenced with either CX-4945 monotherapy, venetoclax monotherapy, a combination therapy of CX-4945 and venetoclax (Combo), or a vehicle-only control. Upon disease manifestation (on day 28 after tail vein injection of cells), spleens and bone marrow were collected from each group for comprehensive evaluation, encompassing the visual inspection (a) and the measurement of spleen weight (b). The spleen (c) and bone marrow cells (d) were immunostained with human-specific CD45 and CD7 antibodies to determine the frequency of immature cells. Body weight fluctuations among the mice were tracked and subjected to statistical analysis (d). For survival assessment, mice that were not harvested for tissue were followed, and survival data were plotted using the Kaplan-Meier estimator (e). Survival differences were statistically evaluated using the Chi-square test.

The findings revealed that the CX-4945 and venetoclax combination therapy exhibited a significantly enhanced therapeutic effect in the PDX model compared to single-agent treatments. This combination therapy led to a substantial reduction in spleen weight (Combo vs. CX-4945: 0.11 ± 0.038 g vs. 0.61 ± 0.095 g, *p* < .001. Combo vs. Venetoclax: 0.11 ± 0.038 g vs. 0.33 ± 0.063 g vs. *p* < .001) (Figure 7a) and a marked decrease in the percentage of leukemia cells in both the spleen (Combo vs. CX-4945: 23.60 ± 6.43% vs. 68.13 ± 3.79%, *p* < .001. Combo vs. Venetoclax: 23.60 ± 6.43% vs. 67.27 ± 5.06% vs. *p* < .001) (Figure 7b & S2A) and bone marrow (Combo vs. CX-4945: 11.57 ± 2.14% vs. 72.32 ± 5.37%, *p* < .001. Combo vs. Venetoclax: 11.57 ± 2.14% vs. 51.30 ± 4.63% vs. *p* < .001) (Figure 7c & S2B).

Throughout the experiment, we monitored the mice’s body weight, and the results showed no adverse impact on weight from either CX-4945, venetoclax, or the combination therapy (*p* > .05).

Survival analysis using the Kaplan-Meier method further demonstrated that the combination of CX-4945 and venetoclax significantly extended survival compared to monotherapy with either agent (Combo vs. CX-4945: *p* = .0008, Combo vs. Venetoclax: *p* = .0005) (Figure 7D).

In conclusion, these results confirm that the combination of CX-4945 and venetoclax provides superior therapeutic efficacy in the T-ALL PDX model.

## 4. Discussion

T-ALL is a highly heterogeneous disease, which can be categorized into four distinct stages: pro-T (CD7+, CD2-, CD5-), pre-T (CD7+, CD2+, CD5±), cortical-T (CD1a+), and mature T (sCD3+, CD1a-).^19^ The conventional T-ALL treatment regimen involves intensive therapy with multiple drugs, however, there is still a significant gap in achieving complete cure for T-ALL, especially in ETP-ALL.^20–22^ Therefore, the development of novel targeted therapeutic strategies and the improvement of prognosis, especially in adult T-ALL patients, is particularly urgent.

IKZF1 acts as a tumor suppressor in T-ALL,^23,24^ and haploinsufficiency stemming from gene mutations leads to an unfavorable prognosis in T-ALL patients.^25^ In our study, we have shown that CX-4945 can restore the repressive function of IKZF1 as a transcriptional regulator of *BCL-2*. In B-ALL cells expressing the dominant-negative Ikaros isoform 6 (IK6), the effect of CX-4945 in inducing *BCL-2* expression is significantly diminished. In contrast, in B-ALL and T-ALL cells lacking the dominant-negative Ikaros isoform, CX-4945 is observed to significantly induce *BCL-2* expression. These results suggest that the effect of CX-4945 in inducing *BCL-2* expression is mediated by IKZF1, and the presence of IK6, which strongly inhibits IKZF1 function, significantly mitigates this effect. This repressive effect is also partially facilitated by chromatin remodeling, as evidenced by the increased enrichment of the repressive histone mark H3K27me3^26^ at the *BCL-2* promoter upon *IKZF1* overexpression, whereas *IKZF1* downregulation results in reduced H3K27me3 enrichment. Furthermore, the combination of CX-4945 with the *BCL-2* inhibitor venetoclax exerts a synergistic effect in T-ALL. The data presented here unveil a novel therapeutic approach in T-ALL by modulating *BCL-2* expression.

Evasion of apoptosis is a hallmark of human malignancies,^27,28^ typically mediated by pro-survival BCL-2 family proteins, which play a pivotal role in acute lymphoblastic leukemia (ALL) and contribute to the paradox of apoptosis in cancer, as well as being a target for therapeutic intervention.^27–29^ In T-ALL, the evasion of apoptosis also determines the resistance to many chemotherapeutic agents.^29^ BCL-2 family members have emerged as attractive therapeutic targets in T-ALL, as they dictate whether T cells undergo apoptosis or migrate to peripheral tissues.^30–32^ *BCL-2* is frequently overexpressed in T-ALL,^20,33^ and in early T-cell precursor (ETP)-ALL, leukemia blasts overexpress and rely on the anti-apoptotic protein BCL-2.^34^

Metabolic changes also provide cancer cells with an advantage in surviving difficult conditions and proliferating uncontrollably. Targeting cellular metabolism is emerging as a promising strategy in cancer treatment. In T-ALL, oxidative phosphorylation (OxPhos) is a critical pathway for leukemia cell survival,^35^ and there is a direct relationship between NOTCH1, elevated OxPhos gene expression, and acquired chemoresistance in pre-leukemic and leukemic models.^36^ Additionally, ETP-ALL exhibits distinctive regulation of ATP synthesis coupled with electron transport and OxPhos,^37^ suggesting that targeting OxPhos offers a potential therapeutic direction for T-ALL. The Bcl-2 protein has been reported to regulate mitochondrial metabolism, leading to reduced ATP and ROS production, including through OxPhos.^38^ Previous studies have shown that in acute leukemia, leukemia stem cell (LSC) populations often exist in a quiescent cell cycle state with low energy production, relying more on oxidative phosphorylation than glycolysis for energy generation.^39^ These LSCs tend to express higher levels of BCL-2. Inhibiting BCL-2 can target oxidative phosphorylation and selectively eliminate leukemia stem cells. Collectively, targeting BCL-2 to clear LSCs and treat T-ALL holds significant clinical potential. As emphasized in our study, dual targeting of BCL-2 effectively eliminates leukemia cells in both in vitro and in vivo experiments. In future research, it will be important to further investigate the effects of BCL-2 inhibition on the OxPhos metabolism of LSCs in T-ALL, providing additional evidence for clinical application.

Venetoclax is a small molecule that specifically targets BCL-2,^40,41^ demonstrating a synergistic effect when combined with cytarabine *In vitro* for the treatment of T-ALL.^20^ Similar to its applications in AML, early Phase I clinical trials in T-ALL have also indicated the emergence of resistance to venetoclax.^33^ The binding affinity between anti-apoptotic proteins and venetoclax is crucial for its function, as point mutations in the BCL-2 protein’s BH3 binding domain can mediate the development of secondary venetoclax resistance, such as the Gly101Val mutation.^42^ In CLL, the Gly101Val mutation in BCL-2 (where valine replaces glycine at position 101 of the BCL-2 protein) results in a significant reduction in the affinity of venetoclax for BCL-2, by approximately 180-fold.^42^ Post-translational modifications of BCL-2 and its related family members, such as phosphorylation, may prevent venetoclax from displacing BIM on BCL-2, thereby blocking the mitochondrial-mediated apoptotic pathway induced by venetoclax.^40,43–45^ This has been confirmed in follicular lymphoma.^40,43,44,46^ Mono-venetoclax treatment-induced drug resistance necessitates additional therapeutic strategies. Combination therapies based on venetoclax to treat T-ALL have garnered interest, such as venetoclax in combination with cytarabine.^45,47,48^ In this study, we discovered that IKZF1 inhibition by CX-4945 could reduce the transcriptional level of BCL-2, highlighting a novel strategy for overcoming drug resistance due to BCL-2 binding domain alterations and modifications. Another interesting finding is that, we also identified that CX-4945 not only diminishes BCL-2 transcription in an IKZF1-dependent manner but also leads to the downregulation of MCL-1 expression. Previous research has indicated that CX-4945 can facilitate the degradation of MCL-1 by reducing protein synthesis, thereby enhancing apoptosis.^49^ Our findings provide further evidence supporting this mechanism. Moreover, the synergistic effect of CX-4945 in combination with Venetoclax in T-ALL cells is partially mediated by the reduction of BCL-2 and MCL-1 protein levels, which in turn promotes apoptosis in T-ALL cells.

The data presented in this study demonstrate that dual targeting of *BCL-2* at both the transcriptional and protein levels in T-ALL elicits a synergistic anti-leukemic effect (Figure). In both CEM- CDX and ETP- PDX xenograft models, the combination therapy significantly reduced tumor burden, as evidenced by decreased spleen weight and reduced leukemia cell infiltration in the spleen and bone marrow. Concurrently, this combined approach extended the survival of CDX and PDX mice without exacerbating side effects. These findings suggest that the co-administration of CX-4945 and venetoclax results in a deeper remission of disease in T-ALL, including ETP-ALL.

**Figure 8.**
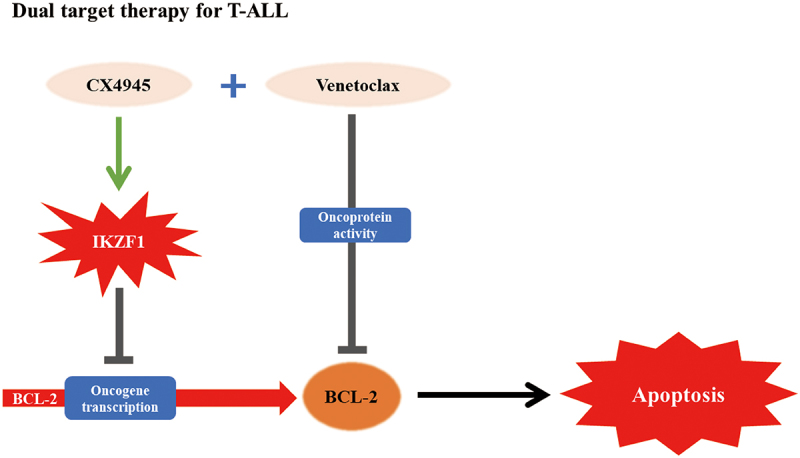
Molecular mechanism model of dual targeting therapy for T-ALL.

In conclusion, our study introduces a novel combination treatment strategy for T-ALL by targeting BCL-2 at two levels (Figure 8). This innovative approach, which involves modulating the transcriptional expression of BCL-2 alongside the specific inhibition of the corresponding oncoprotein, may offer a new therapeutic option for the treatment of T-ALL.

## Supplementary Material

Supplementary methods tables and figures.docx

## Data Availability

The datasets used and/or analyzed during the current study available from the corresponding author on reasonable request.
