# Supplementary material for: Targeting the IKZF1/BCL-2 axis as a novel therapeutic strategy for treating acute T-cell lymphoblastic leukemia
Source: Cancer Biol Ther. 2025 Jan 25;26(1):2457777. doi: 10.1080/15384047.2025.2457777 (PMC11776473; doi:10.1080/15384047.2025.2457777)
Supplement: Supplementary methods tables and figures.docx [file KCBT_A_2457777_SM0015.docx]

**Supplemental Information**

**Targeting the IKZF1/BCL-2 Axis as A Novel Therapeutic Strategy for Treating Acute T-Cell Lymphoblastic Leukemia**

Juan Li^1,2^, Chunmei Ye^1,2^, Hui Li^1^, Jun Li^1,2,^*

^1^Department of Hematology, Taixing People’s Hospital Affiliated to Yangzhou University, Taixing, China.

^2^Institute of Hematology, affiliated hospital of Yangzhou University, Taixing, China.

Running title: Targeting IKZF1/BCL2 signaling in T-ALL

*Correspondence to:

Jun Li, M.D., Ph.D.

Department of Hematology

Taixing People’s Hospital, Affiliated hospital of Yangzhou University

1 Changzheng Road, Taixing 225400, China,

E-mail: lijun408006@126.com

The authors declare no conflicts of interest

**Supplemental methods**

**CUT&Tag Assay Kit for sequencing**

The CUT&Tag protocol was performed according to the manufacturer’s instructions. Briefly, MOLT-4 cells were harvested and incubated with IKZF1 antibody overnight at 4°C. Each sample was then mixed with 10 μL of activated concanavalin A-coated magnetic beads (Vazyme, China) and incubated at room temperature for 15 minutes. The bead-bound cells were resuspended in 100 μL of Dig-Wash Buffer containing 2 mmol/L EDTA and a 1:50 dilution of anti-NrF2 antibody, followed by overnight incubation at 4°C. Subsequently, a secondary antibody (1:50 dilution) was added and incubated for 30 minutes at room temperature.

A pA-Tn5 adapter complex (~0.04 μmol/L) was gently vortexed into the samples and incubated for 1 hour at room temperature. The samples were then resuspended in Tagmentation Buffer and incubated at 37°C for 1 hour. Following this, DNA extraction and proteinase K digestion were carried out, and the samples were purified using Ampure XP beads. CUT&Tag libraries were amplified via PCR using the PCR Master Mix (Vazyme, China), and the quality of the resulting libraries was assessed using an Agilent 2100 Bioanalyzer (Agilent Technologies, Japan).

**Plasmid construction, lentiviral transduction, and target** **gene Knockdown or overexpression**

Lentiviral shRNA plasmids for *IKZF1* were constructed by subcloning the shRNA oligos into the lentiviral shRNA vector with an IRES GFP (pLV3ltr-ZsGreen-Puro-U6) (Corues Biotechnology, China) or overexpression vector (pCDNA3.1-EF1a-ZsGreen1-SV40-Puro) (Corues Biotechnology, China) as the manufacturer’s instruction.

293T cells were transfected with the indicated viral plasmid and packaging plasmids (psPAX2 and pMD2.G) (Corues Biotechnology, China), via Exfect Transfection Reagent (Vazyme Biotech Co., Ltd, China, NO. T101-01), following the manufacturer’s instructions. Viral Supernatant was collected and filtered with a 45μM filter 48- and 72- hours post-transfection. Cells were then transduced with the resulting lentivirus. Protamine sulfate (Beyotime Biotechnology, China) was added to increase the transduction efficiency. 48 hours post-transduction, infected cells were treated with 1μg/mL puromycin (InvivoGen, CAS.58-58-2) for stable expressing cell selection. The efficiency of transduction was monitored by GFP (+) cells via flow cytometry, and more than 95% of GFP (+) cells were used for knockdown efficiency and further functional analysis.

**T-ALL cell line derived xenograft model (CDX) mouse models**

Four-week-old male NOD/ShiLtJGpt-Prkdc^em26Cd52^Il2rg^em26Cd22^/Gpt (NCG) mice were purchased from GemPharmatech Corporation and housed in a specific pathogen-free facility. A pilot assay was conducted to determine the appropriate inoculated CEM cell numbers and corresponding CX-4945 doses based on mouse survival. For the main experiment, 2×10⁵ CEM cells per mouse were injected intravenously into the NCG mice. For the combination treatment study, CX-4945 was dissolved in a 25mM Na₂HPO₄ solution to reach a concentration of 15 mg/ml. Following engraftment, mice were divided into four groups and received daily treatments: vehicle control, CX-4945 via gavage at 100 mg/kg, venetoclax via intragastric administration at 50 mg/kg, or the combination of both CX-4945 and venetoclax.

Post-treatment, bone marrow (BM) and spleen cells were collected from euthanized mice, and red blood cells were removed using RBC lysis buffer (Biosharp, China). To assess leukemia burden in the BM and spleen, the remaining cells were stained with human-specific CD45 antibodies (PerCP anti-human CD45, BioLegend, Cat:368506) for 30 minutes and analyzed by flow cytometry (Beckman). All experimental procedures were approved by the Animal Care Committee of Southeast University and adhered to the Regulations for the Administration of Affairs Concerning Experimental Animals in China.

**T-ALL patient-derived xenograft (PDX) mouse models**

For the primary human T-ALL mouse xenograft model, a pilot assay was conducted to determine the optimal inoculated cell numbers and appropriate drug dosages for each patient sample. In the main experiment, 5×10⁵ patient-derived T-ALL cells were transplanted intravenously into NCG mice as described previously. CX-4945 was dissolved in a 25mM Na₂HPO₄ solution to a concentration of 15 mg/ml, while the BCL2 inhibitor venetoclax was prepared in a solution of 5% DMSO, 40% PEG300, 2% Tween80, and 53% sterilized water.

Following engraftment, the mice were randomly assigned to four groups: vehicle control (Group 1), CX-4945 administered daily via gavage at 100 mg/kg (Group 2), venetoclax given once daily via intragastric injection at 50 mg/kg (Group 3), and combination treatment with both CX-4945 and venetoclax at the same doses as in the single-drug groups (Group 4). After the treatment period, bone marrow (BM) and spleen cells were harvested from euthanized mice, and red blood cells were lysed using RBC lysis buffer (Biosharp, China).

To quantify leukemia burden in the BM and spleen, cells were stained with human-specific CD45 (PerCP anti-human CD45, BioLegend, Cat:368506) and CD7 (PE anti-human CD7, BD, Cat:663533) antibodies for 30 minutes and analyzed using flow cytometry (Beckman). Additionally, immunohistochemical analysis was performed using a human CD45 antibody (Proteintech, Cat: 20103-1-AP). All experimental procedures were approved by the Animal Care Committee of Yangzhou University and complied with China's Regulations for the Administration of Affairs Concerning Experimental Animals.

**Bioinformatic analysis of Cut &Tag sequencing data**

Sequencing quality was assessed using FastQC software (http://www.bioinformatics.babraham.ac.uk/projects/fastqc/). Low-quality bases and adaptors were trimmed using Trimmomatic, retaining only reads of at least 50 bp in length for further analysis. The reference genome and gene annotation files were obtained from the UCSC Genome Browser (http://genome-euro.ucsc.edu/). Paired clean reads were aligned to the reference genome using Hisat2 v2.0.5.

To explore and visualize large-scale genomic data, we utilized the Integrative Genomics Viewer (IGV) from the Broad Institute. The R scripts used for transcriptomic data analysis are publicly available on GitHub (https://github.com).

**Supplementary tables**

**Table S1 Primer sequences**

|  | Forward | Reverse |
| --- | --- | --- |
| BCL-2 | 5'-GACTTCGCCGAGATGTCCAG-3' | 5'-GAACTCAAAGAAGGCCACAATC-3' |
| IKZF1 | 5'-TACCTGACCAACCACATCGC-3' | 5'-GCGTTATGTGCGACGAGAAC-3' |
| GAPDH | 5'-GCAAATTCCATGGCACCGT-3' | 5'-GACTCCACGACGTACTCAGC-3' |

**Table S2 Oligo sequences design for scramble RNA of *IKZF1***

|  | Forward | Reverse |
| --- | --- | --- |
| BCL-2 | 5'-GCGGAGGATTTACGAATGCTT-3' | 5'-AAGCATTCGTAAATCCTCCGC-3' |

**Supplementary Figures**

**Figure S1**

**
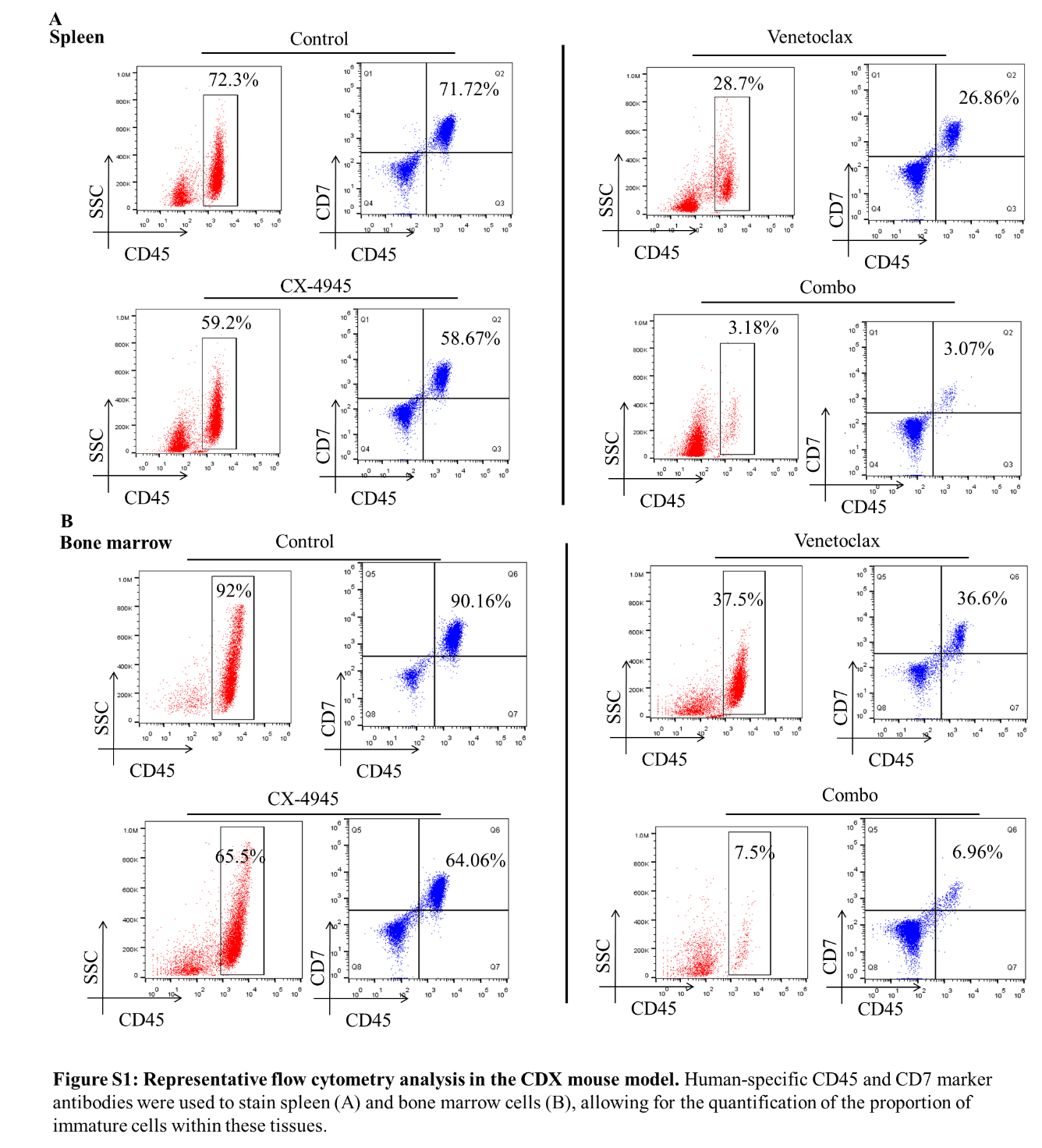
**

**Figure S2
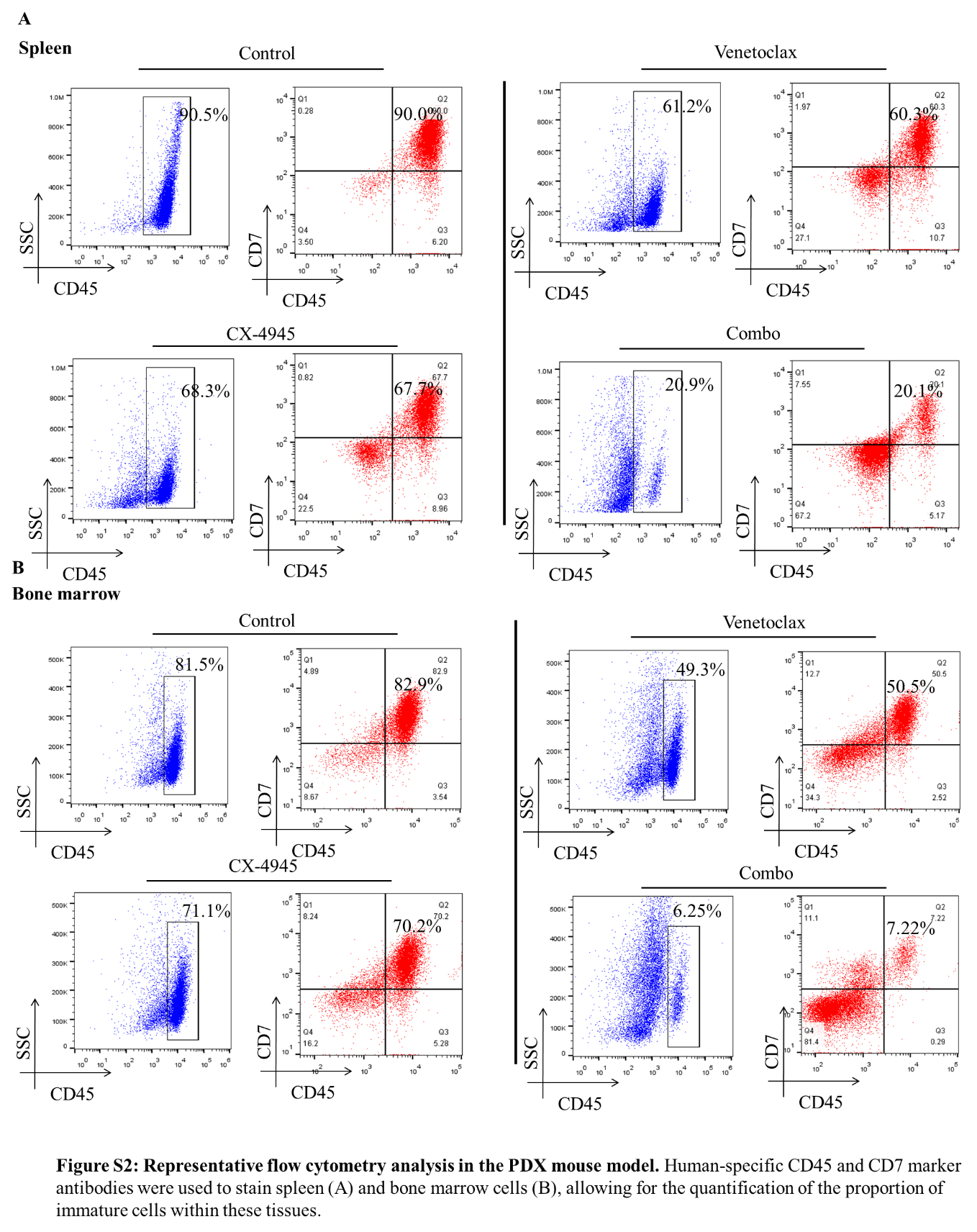
**
